# Marketing Breast*feeding* Substitutes: A Discussion Document

**DOI:** 10.3390/ijerph17249239

**Published:** 2020-12-10

**Authors:** Genevieve E Becker

**Affiliations:** BEST Services, Galway, Ireland; gbecker@bestservices.ie

**Keywords:** marketing, code, breastfeeding, protection, breast pump

## Abstract

Marketing influences knowledge, attitudes, and decisions related to infant and young child nutrition, safety, development, parental confidence, and other aspects of health and wellbeing of the child. These attitudes and behaviours of parents, health workers, policy makers, and other influencers have short- and long-term effects on the child. There is an International Code of Marketing of Breast-Milk Substitutes. Is it time to have a code of marketing of breast*feeding* substitutes?

## 1. Introduction

In 1981, the World Health Assembly (WHA) passed the International Code of Marketing of Breast-Milk Substitutes, which sought to protect breastfeeding and reduce the damage due to inappropriate marketing of breast-milk substitutes, feeding bottles, and artificial teats. This Code has been subject to regular review and subsequent resolutions by the World Health Assembly [1].

Its title “Breast-Milk Substitutes” puts the focus on the milk, or the nutritional product. The physical act of breastfeeding is much more than fuelling a baby with milk, it also includes the physiological norm. The action of breastfeeding provides comfort and connection [2], builds a well-functioning immune system [3,4], reinforces innate appetite control [5], develops strong orofacial muscles, and contributes to speech, dental, and respiratory health [6,7], in addition to the nutritional components—effects that are specific to the act of breastfeeding. Breastfeeding also affects the mother’s psychological, nutritional, hormonal, and biochemical state [8], which in turn influences the child’s health and wellbeing. 

Over the years, the increase in marketing and sales of devices and equipment that impair this connection, or seek to separate the mother and baby as one unit into separate parts, is undermining the important holistic aspects of breastfeeding. These devices and equipment include breast pumps, nipple shields and extenders; sleep and soothing devices such as pacifiers, automatic rockers, and cribs, among other devices. This article focuses on one example of the devices that may substitute for breastfeeding—breast pumps—and their marketing. The world breast pump market was valued at USD 540 million in 2015 and is projected to grow by 6.6% to reach USD 829 million by 2022 [2]. 

Organisations and individuals have raised concerns about the marketing of breast pumps for many years [9,10,11,12,13,14,15,16,17]. Perhaps it is time to create a code of marketing of breast*feeding* substitutes. 

## 2. Influence of Marketing Activity

Marketing activity is defined as product promotion, distribution, selling, advertising, product public relations, and information services [18]. Marketing is more than advertising; it uses psychology to identify needs and wants and then to increase acceptability of “solutions” to those needs and wants. 

Marketing may provide information promoting the value of pumping milk. This information is provided for health workers and parents as well as the general community, through educational events, celebrity endorsements and social media influencers, media, and product positioning. Marketing effect spreads from person to person and health professionals may in turn influence other health professionals to develop “a relationship with a [pump] rental station or dealer. They should then provide women with the name of a specific breast pump just as they write prescriptions for a specific drug as well as exact information about where to obtain the pump” [19]. The potential negative effect on infant health of relationships between health workers and commercial entities was recognised in Article 7.3 Code of Marketing of Breast-Milk Substitutes and subsequent WHA resolution 49.15 [1].

Companies may lobby decision makers and fund “experts” to assist with guidelines for government programs, as well as employer and professional organisations, such guidance reinforces the position that pumping milk is comparable to breastfeeding. For example, provision or reimbursement by health insurance for a breast pump for all new mothers is mandated by the USA Affordable Care Act [20]; however, paid maternity leave, which values the holistic relationship between child and mother of which the provision of milk is only one aspect, is not nationally mandated. Attitudes toward the dual role of mothering a societies’ healthy future and women’s role in economic employment in some societies has resulted in early separation and short breastfeeding. A breast pump is seen as the solution—human milk is valued, but as a product, not as a relationship. Some elements of workplace policies, which claim to support breastfeeding, are actually supporting the use of a breast pump [21]. 

Marketing influences the knowledge and attitudes of pregnant women and new mothers, of her family and friends, and of the general public, as well as the attitudes of the health workers and health services that educate and advise. This influence was recognised in the existing Code of Marketing of Breast-Milk Substitutes [1] by calling for the prohibition of contact between breast-milk substitute companies, distributors, or personnel and pregnant women, new mothers, and their families. Marketing of breast-milk substitutes created attitudes that their use was the norm; marketing may similarly create the attitude that pumped milk given in a bottle is more acceptable outside the home than feeding at the breast or that fathers feel left out or have no bond if they do not feed their baby [14]. A UK National Health Services (NHS) poster shows a father bottle feeding a baby with the text: “Sue expresses milk so I can help with feeds …” [21]. This public health message ignores the additional chore the mother needs to take on to express her milk for the father to “help”, as well as encouragement to purchase and regularly use a breast pump. Images used in breast pump advertisements may show father–infant bonding through feeding pumped milk in a bottle, and show the mother and baby in intimate and close contact, which is head to head, with the breasts themselves cropped out of the image suggesting that “breastfeeding is unnecessary to infant feeding and unnecessary to bonding and ultimate happiness for the child and for the mother” [22].

## 3. Effects of Marketing on Behaviours

Devices to remove milk from the breast date back centuries, though only used by a minority of women, and the need to remove milk may have been met previously by women learning the skill of hand expression. However, there is little commercial profit from this skill, and it is less visible than using a breast pump. This low visibility of hand expression and the high visibility of pumps due to marketing has resulted in the situation where data collection on infant feeding may not include hand expression as a response option [23], or may exclude mothers who reported hand-expressing milk only [24,25], and there may be ambiguity about the meaning of the term breastfeeding [14,26,27,28]. Research activity and funding may use the terms milk expression and pumping interchangeably in publications with no definition, though physiologically and psychologically they are distinct [21,29]. Health worker training, guidance, and practice may present the use of a pump as the norm and hand expression as an alternative if a pump is not affordable or available, if hand expression is mentioned at all [30,31].

Once the attitude or belief has been created, that there is a problem and thus there is a need for a solution, that pumps are an essential item to purchase for newborn care rather than an exception, the other aspects of the marketing process can then build the market share for the product. Maternal confidence and the relational aspects of breastfeeding may be undermined when women’s bodies are viewed like machines producing a product (milk) supplied to a consumer (the baby) [32]. Pumps may be provided free to maternity units, which may undermine mother’s confidence by encouraging reliance on technology instead of on the baby or the mother’s own body [33]. Health workers may see using a pump as a quick solution to newborn breastfeeding challenges rather than spending time with the mother and baby to address the challenge. Mothers have reported that pumping facilitates greater control over infant feeding and reassurance that the baby was getting enough milk [33]. Similar to studies that indicate in-hospital pacifier use may be a marker for lack of confidence or issues with mothering [34], seeking a pump for reassurance and a sense of control may be a marker for needing additional support. 

## 4. Insufficient Information 

Mothers may use a breast pump for a broad range of reasons [35] and a mother’s decision to use a breast pump is to be respected. However, if information predominately comes from marketing by pump manufacturers and distributors, then that decision may not be based on full, accurate, and scientific information. Information on pumping may be conflicting and inconsistent. Commercial websites may identify separation from the baby and pumping as a positive choice of benefit to the parents and wider family and emphasise difficulties with direct breastfeeding [36]. There may be little information on the time, energy, and financial costs of purchasing a pump, attachments, and milk storage containers; the hygiene needed; the storage required; and the time to set-up, pump, and clean-up. The cost of obtaining equipment may limit access and increase health inequalities for vulnerable infants [37,38].

Rarely does marketing information include any possible risks from using pumps, though problems and adverse effects have been reported [14,15,25,30,39,40]. These reports include injuries such as damaged nipples and breast tissue, bruising, pain, skin irritation, muscular-skeletal issues, and contamination of breastmilk, as well as problems with the volume of milk extracted. The need for accurate information on the risks of using substitutes is recognised in the existing Code of Marketing of Breast-Milk Substitutes [1].

There are indications that pumped milk may differ in some nutrients from expressed milk. These differences may be important for preterm infants and possibly for infants totally dependent on expressed/pumped milk [35]. Feeding pumped milk by bottle and teat is associated with reduced appetite control and over-consumption of milk, resulting in higher risk of overweight than when feeding directly at the breast [5].

Electricity is also required for using many of the pump types, making their use unreliable in situations of power outages, natural disasters, and emergencies. Pump equipment contains plastics, batteries, precious and heavy metals, as well as packaging materials, which ultimately end up creating waste disposal concerns. Information on pump equipment rarely includes environmental information to allow parents, health workers, and health policy makers to make informed decisions on the broad and far reaching environmental aspects of reliance on pumped milk [41].

## 5. Protection

Consumer protection regulations aim to ensure safe products, to prevent deceptive advertising, to facilitate redress for breaches of consumer rights, and to ensure appropriate, accurate, and truthful information is provided to make informed product choices. There are international systems for monitoring consumer protection law and rights, though they may depend on individual consumers or consumer organisations to know the rights and regulations, to report breaches of regulations, and to follow-up to receive the protection [42]. The manufacture and sale of medical devices may be regulated, including manufacturing standards, registration of devices, and reporting of adverse effects of their use [43,44,45,46]. However, the definition of a medical device may differ, for example, a breast pump may be defined as a consumer item in one country and a medical device in another country.

Although these regulations may focus on the safety of the device, the marketing of the device is what influences attitudes. One of the aims of the International Code of Marketing of Breast-milk Substitutes [18] is protection from inappropriate marketing—protection for the baby, the mother, the family, and health workers.

When breastfeeding and nurturing at the breast is recognised as the unequalled way to nourish and care for an infant and to provide a unique biological and emotional basis for health and wellbeing of the child and the mother, it will then be important to protect this practice.

Awareness of and possibly restrictions on infant and young child related marketing serves to protect infants and young children, their parents, and health workers. One way to protect breastfeeding would be to develop, agree, implement, monitor, and enforce a code of marketing for breast*feeding* substitutes similar to the International Code of Marketing of Breast-Milk Substitutes (1981).

## 6. Conclusions 

Marketing is designed to affect behaviour. Care-giver behaviours have short- and long-term influences on child health and wellbeing. This paper offers a starting point for discussions to develop, agree, implement, monitor, and enforce a code of marketing of breastfeeding substitutes that might be situated separately or as an addition to the existing International Code.

Extending the existing International Code of Marketing of Breast-Milk Substitutes [1] and gaining agreement from the over 100 countries that form the World Health Assembly would take many years to achieve. However, a health provider does not need to wait for an agreed international code; they can use the attached checklist (Appendix A) to assess and highlight marketing activities that can indicate their protection and support for breastfeeding and to offer ideas for improvements.

## Appendix A

**Code of Marketing of Breast*feeding* Substitutes—what might it look like?**

*A discussion Document*

The aim of this Code of Marketing of Breast*feeding* Substitutes is to contribute to the provision of safe and adequate care and nutrition for infants by the protection, support, and promotion of breastfeeding and nurturing at the breast and by ensuring the proper use of breastfeeding substitutes, when this is necessary, on the basis of adequate information and through appropriate marketing and distribution of devices.

Action areas:

- Information and education
- Marketing to the general public and to mothers
- Health care systems
- Health workers
- Persons employed by manufacturers and distributors
- Labelling
- Quality
- Implementation and monitoring

**Checklist for Marketing of Breast*feeding* Substitutes**

*How does your health facility or practice rate?*

1. Does any information or education materials provided on infant care or feeding show a device company brand or logo or contain an image that may idealise the use of that device? If yes, are these educational materials
  - produced or sponsored by manufacturers of breastfeeding-related devices?
  - created or provided from within the health service?
2. Do the information or instructional materials needed for the use of a device include information on the importance of breastfeeding and nurturing at the breast, possible negative effects of use of the device, and how to safely and effectively use and clean the device?
3. Do pregnant women, new mothers, or mothers of hospitalised infants receive samples of breastfeeding-related devices?
4. Do pregnant women, new mothers, or mothers of hospitalised infants, or their families, receive discount, money off, or special offers for the purchase of breastfeeding related devices?
5. Do pregnant women, new mothers, or mothers of hospitalised infants receive gifts (e.g., toys, baby items, and mother items), which are donated, produced, or sponsored by manufacturers or distributors of breastfeeding related devices? If yes, do these gifts carry product or company identification, logos, etc.?
6. Do personnel from manufacturers or distributors of breastfeeding related devices have any contact with individual or groups of pregnant women, new mothers, mothers of hospitalised infants, or their families at the health facility?
7. Are any displays, posters, or equipment visible to pregnant women, new mothers, mothers of hospitalised infants, and their families that encourage or promote the use of a specific brand of breastfeeding or infant-care-related device?
8. Are any displays, posters, or equipment that encourage or promote the use of a specific brand of breastfeeding or infant-care-related device visible to or distributed to staff members/health workers?
9. Are any breastfeeding or infant-care-related devices given to the health facility free or at subsidised cost?
10. Are any training or education sessions provided to staff members/health workers by personnel from manufacturers or distributors of breastfeeding or infant-care-related devices restricted to scientific and factual matter specific to the use of their device?
11. Do staff members/health workers receive information on the possible risks to the mother, infant, or breastfeeding from use of any breastfeeding or infant-care-related devices present in the health facility?
12. If use a breastfeeding related device is recommended by a staff member/health worker of the health facility, is there a system in place to provide follow-up care by a suitably qualified person?
13. If a suitably qualified staff member/health worker of the health facility recommends that a mother/infant use a specific breastfeeding-related device, is there a system to provide this device free of charge for as long as the need for the device continues?
14. Does the health facility limit choice of which manufacturers of breastfeeding-related devices will provide products for use in the health facility? If yes, is there a transparent policy or process to decide which manufacturers’ products are used and how procurement is reviewed?
15. Do manufacturers or distributors of breastfeeding-related devices give staff members/health workers product samples, gifts, free events, sponsorship, study tours, conference attendance, or products for the personal use of the staff member/health worker or their family? If yes, is the staff member/health worker required to disclose such funding and gifts to their employer? If yes, are manufacturers or distributors of breastfeeding-related equipment required to disclose funding or gifts to a staff member/health worker of the health facility?
16. Does the labelling of the device list the materials used in the manufacture and distribution of the device and how they can be recycled or disposed of after use?
17. Does the labelling of the device indicate if there is a quality and safety standard or device registration applicable to the device?
18. Are the label and instructions for use shown in the language(s) commonly used in the area?
19. Is the name of the manufacturer apparent on the individual device if the packaging for the device is removed?
20. Are the name and contact details of the manufacturer, and local distributor if relevant, included on the device or on its accompanying instruction material?
21. Are there any labelling or packaging images that idealise the use of the device, suggest the use of the device is equivalent or superior to breastfeeding or nurturing at the breast, or discourage or undermine breastfeeding?
22. Is there a written policy in the health facility describing the allowable and prohibited contacts with marketing personnel from breastfeeding related device companies; the acceptance or not of donations, sponsorship, or other gifts by staff members of the health facility; the provision of samples or gifts to mothers by the companies; or the provision of educational materials carrying company identification?
23. Is regular monitoring carried out to ensure the health facility’s practices do not serve to market breastfeeding related devices?

- What practices indicate the health facility’s protection for breastfeeding and should be supported to continue?
- What practices need to change to better protect breastfeeding? What is needed to change these practices?
